# Human and Canine Echinococcosis Infection in Informal, Unlicensed Abattoirs in Lima, Peru

**DOI:** 10.1371/journal.pntd.0001462

**Published:** 2012-04-03

**Authors:** Maria M. Reyes, Claudia P. Taramona, Mardeli Saire-Mendoza, Cesar M. Gavidia, Eduardo Barron, Belgees Boufana, Philip S. Craig, Luis Tello, Hector H. Garcia, Saul J. Santivañez

**Affiliations:** 1 Alberto Hurtado School of Medicine, Universidad Peruana Cayetano Heredia, Lima, Perú; 2 School of Veterinary Medicine, Universidad Nacional Mayor de San Marcos, Lima, Perú; 3 School of Environment and Life Sciences, University of Salford, Salford, United Kingdom; 4 Instituto Peruano de Parasitología Clínica y Experimental, Lima, Peru; 5 Department of Microbiology, School of Sciences, Universidad Peruana Cayetano Heredia, Lima, Peru; 6 Center for Global Health - Tumbes, Universidad Peruana Cayetano Heredia, Lima, Peru; 7 Cysticercosis Unit, Instituto Nacional de Ciencias Neurologicas, Lima, Peru; Asahikawa Medical College, Japan

## Abstract

*Echinococcus granulosus* infections are a major public health problem in livestock-raising regions around the world. The life cycle of this tapeworm is sustained between dogs (definitive host, canine echinococcosis), and herbivores (intermediary host, cystic hydatid disease). Humans may also develop cystic hydatid disease. Echinococcosis is endemic in rural areas of Peru; nevertheless, its presence or the extension of the problem in urban areas is basically unknown. Migration into Lima, an 8-million habitant's metropolis, creates peripheral areas where animals brought from endemic areas are slaughtered without veterinary supervision. We identified eight informal, unlicensed abattoirs in a peripheral district of Lima and performed a cross-sectional study in to assess the prevalence of canine echinococcosis, evaluated by coproELISA followed by PCR evaluation and arecoline purge. Eight of 22 dogs (36%) were positive to coproELISA, and four (18%) were confirmed to be infected with *E. granulosus* tapeworms either by PCR or direct observation (purge). Later evaluation of the human population living in these abattoirs using abdominal ultrasound, chest X-rays and serology, found 3 out of 32 (9.3%) subjects with echinococcal cysts in the liver (two viable, one calcified), one of whom had also lung involvement and a strongly positive antibody response. Autochthonous transmission of *E. granulosus* is present in Lima. Informal, unlicensed abattoirs may be sources of infection to neighbouring people in this urban environment.

## Author Summary

Echinococcus granulosus infections are a major public health problem in livestock-raising regions around the world. This parasite is transmitted by dogs, and humans could be accidentally infected, developing cystic lesions in internal organs after several years of infection. The risk of infection has been widely described in Peruvian rural areas; nevertheless the extension of the problem in urban areas is basically unknown. Migration into Lima, an 8-million habitant's metropolis, creates peripheral areas where animals brought from endemic areas are slaughtered without veterinary supervision. In our study, we assess the number of infected dogs, which were living in eight informal, unlicensed abattoirs in a peripheral district of Lima, by evaluation of dog faeces using different techniques. We identified that 4 of 22 dogs were infected with E. granulosus worm. Later evaluation of the human population living in these abattoirs using abdominal ultrasound, chest X-rays and serology, found 3 of 32 subjects had echinococcal cysts in the liver, one of whom had also a cyst in lung and a positive serological test. This work demonstrates that autochthonous transmission of E. granulosus is present in Lima and that informal, unlicensed abattoirs may be sources of infection to neighbouring people in this urban environment.

## Introduction

Canine echinococcosis is caused by the adult stage of the tapeworm *Echinococcus granulosus*; infected dogs are the source of infection for human cystic hydatid disease (CHD), a serious public health problem in farming regions around the world [1], [2]. In the domestic life cycle of *E. granulosus* dogs harbor the intestinal adult tapeworm stage, spreading the parasite' eggs into the environment through their feces. Ruminants (intermediary hosts), ingest infective eggs and develop cysts in their internal organs. Feeding dogs with raw viscera of infected animals contributes to perpetuating this cycle [3], [4]. Humans get infected by accidental ingestion of eggs from tapeworm-infected dogs and develop cystic lesions, principally in liver and lungs, after several years [5]. Both canine echinococcosis and CHD are commonly found in rural farming communities, though there are some reports of human and dog infection in urban areas [6], [7], [8], [9], [10], [11]. In a non-endemic coastal urban city in Peru, a study on abattoir workers and stray dogs from the same areas found 6.25% of canine echinococcosis by examination of the intestinal contents of stray dogs and 12% of human CHD [12].

Lima, the capital of Peru, with a population burgeoning on 8 million people, is assumed to be non-endemic for canine echinococcosis and CHD; the last reported prevalence of canine echinococosis was 0.003% [13]. However, 21% of lung CE patients in a hospital in Lima between 1980 and 1986 were born in the same city and had not spent more than one month in endemic regions [14]. Lima's unique migratory patterns have created regions in the periphery of this city where poor populations bring animals from endemic areas and slaughter them without veterinary supervision. We assessed the prevalence of canine echinococcosis in dogs living in informal, unlicensed abattoirs located in a peripheral district of Lima, and of CHD in the individuals living in the same dwellings.

## Materials and Methods

### Ethics Statement

The protocol and written informed consents were approved by the Animal and Human Ethics Committees of the Universidad Peruana Cayetano Heredia. All subjects older than eighteen years old provided written inform consent; and in the case of children, they provided written inform assent and their parents/guardians provided written consent for them. Animal ethical committee reviewed and approved the protocol according to international guidelines provided by The Office of Laboratory Animal Welfare (A5146-01).

### Study design

Cross-sectional study to determine the presence of canine echinococcosis and human CHD in informal urban abattoirs in Lima, Peru.

### Study area and population

The district of Puente Piedra is one of 49 districts composing metropolitan Lima. Located in the north of Lima, it covers an area of 71.18 km^2^ and has a population density of 3281.35 inhabitants per km^2^ [15]. Based on information collected through interviews to residents of Puente Piedra, we identified ten informal, unlicensed abattoirs where people raise and slaughter cattle and sheep, which are principally brought from endemic areas of the Peruvian highlands.

### Study evaluations - dogs

**Figure 1:**
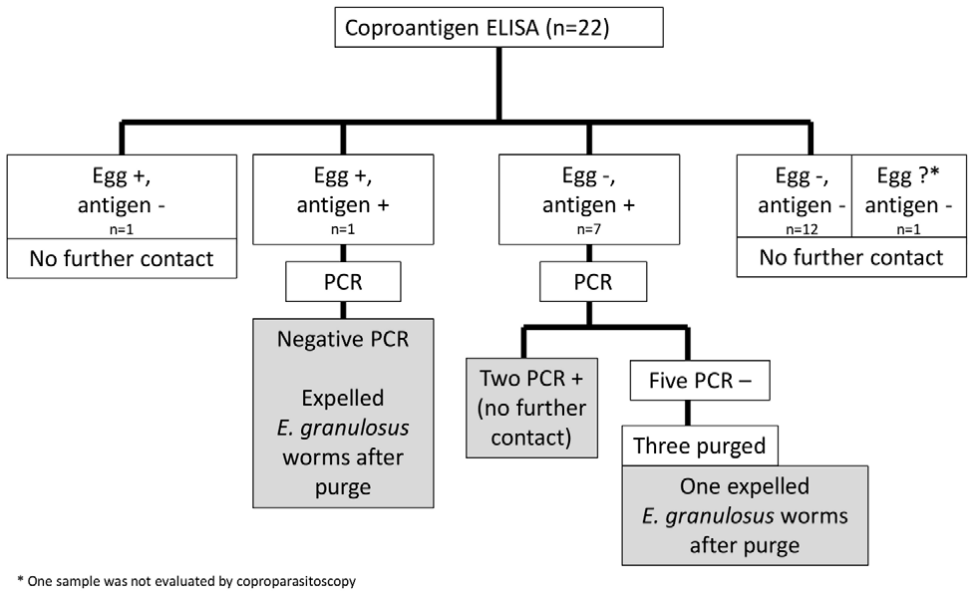
Sequence of evaluations performed in dogs (n = 26).

In each abattoir center we evaluated all dogs older than 2 months that had been living (sleeping and being fed) there for at least 2 months before the visit, excluding dogs recently de-wormed or those that were pregnant.Dog stool samples were evaluated by coproparasitoscopy and coproELISA. Samples positive in coproELISA were evaluated by PCR and the dog had an arecoline bromhydrate purge (Figure 1). A positive dog was defined as any dog with a positive coproELISA, independently of the results of the other evaluations (dogs without a coproELISA evaluation were not included in the analysis). After obtaining the results, praziquantel (one 5 mg/kg dose) was administered to all dogs belonging to abattoir centers where at least one dog was positive by any method. The methods used for each evaluation are briefly described below:

#### I. Coproparasitoscopy

Dog fecal samples (∼4 gms) were placed in PBS Tween 0.3%, and processed according to conventional flotation and sedimentation methods [1]. Each sample was examined microscopically at 10× and 40× amplification. Observation of taeniid eggs in stools is reported as *Taenia* spp. and does not confirm the diagnosis of *E. granulosus* because of the similarity of eggs between cestode species.

#### II. ELISA

The remaining sample volume, also stored in PBS Tween 0.3%, was sent to the University of Salford, UK, for coproantigen detection. A sandwich ELISA technique described by Allan *et al.* (1992) and Craig *et al.* (1995) with minor modifications, according to Lahmar et al (2007), was used [16], [17], [18]. The cut-off value was the mean optical density (OD) of faecal samples from uninfected dogs (controls) plus 3 standard deviations. A cut-off of 0.09 OD units was determined using samples from negative dogs.

#### III. PCR

1 g of fecal material was preserved in 95% ethanol, only from dogs with a positive coproELISA. These samples were also sent to Salford, UK, to be processed by PCR as described by Abbasi *et al.* (2003) with slight modifications in some reagent concentrations [19]. The presence of the diagnostic 133-base pair band marked a positive result.

#### IV. Arecoline purge

4 mg/kg of arecoline bromhydrate was administered to coproELISA positive dogs that could be evaluated. If no effect (defecation) was obtained in 30 minutes, a second dose of 2 mg/kg was given. Post-purge samples were collected, mixed with saline 5% formaldehyde, passed through a sieve, and examined. Helminth worms, including *E. granulosus*, were identified and counted for each dog. The dogs were kept under observation for 2 hours after the purge. All remaining materials were disposed under appropriate biosafety conditions.

Furthermore we evaluated characteristics of dogs (age, weight, gender, feeding habits) and the abattoir location in relationship to the river to determine the association between these variables and the odds of infection in dogs. Information about characteristics of dogs was collected using a questionnaire that was applied to dog's owner.

### Study evaluations – humans

We invited to all subjects older than 3 years olds who were living in the informal abattoirs to be evaluated by abdominal ultrasound (US) and/or chest X-Ray, in addition we offered serological evaluation by Enzyme-linked immunoelectrotransfer blot (EITB). After the evaluations, individuals with abnormal radiological findings were referred to a local health center to be treated. US exams were performed using a Sonosite plus 3.5-MHz portable machine. Each evaluation was video-recorded and sent to a second, different observer to confirm or rule out the diagnosis of CE and its categorization according to the WHO US classification [20]. There were no discrepancies between observers. Posterior-anterior chest x-Rays were taken at a local health center facility and read by a trained radiologist. Human blood samples were obtained by venipuncture and taken to the Center for Global Health laboratories of the Universidad Cayetano Heredia in Lima. EITB was performed as previously described, using purified hydatid cyst fluid [21]. The presence of reactions to one or more of three known antigens (8, 16, and 21 kD) was defined as a positive assay.

### Statistical analysis

χ^2^ tests were used to compare the frequencies of discrete variables. Continuous measurements were presented as median values and compared using Mann-Whitney test. A simple logistic regression (SLR) analysis followed by a multiple logistic regression (MLR) analysis were performed to evaluate the association between individual characteristics and the odds of being infected. A p-value of <0.05 was taken to indicate statistical significance. All analyses were conducted using Stata version 10 (StataCorp LP College Station, TX, USA).

## Results

The owners of 8 out of 10 informal abattoirs in Puente Piedra agreed to participate. From 31 dogs in these abattoir centers, 9 were not evaluated: one was pregnant and for 8 animals fecal samples could not be obtained or were insufficient. Therefore, we analyzed data on 22/31 dogs. Characteristics of evaluated dogs and abattoir centers are presented in table 1. The dogs had a median age of 30 months (range: 4–120), and median weight 16.5 kg (4 to 35). Twelve dogs (54.6%) were male, and only for 4 dogs (18.2%) owners reported feeding them with viscera. Twelve dogs (54.6%) belonged to abattoir centers next to the river.

**Table 1:** Seropisitivity in relation to dog and abattoir characteristics.

Dog/abattoir characteristic	Copro-ELISA positive (n = 8)	Copro-ELISA negative (n = 14)	p value*
**Age (months)**			
Median (range)	27 (8–72)	30 (4–120)	0.630
**Weight (kg)**			
Median (range)	18.5 (6–25)	13.5 (6–35)	0.336
**Gender**			
Male	5 (62.5%)	7 (50.0)	0.571
Female	3 (37.5%)	7 (50.0)	
**Feeding habits of dogs**			
Viscera	1 (12.5%)	3 (21.4)	0.601
Other	7 (87.5%)	11 (78.6)	
**Localization of abattoir**			
Close to river	7 (87.5%)	5 (35.7)	**0.019**
Other	1 (12.5%)	9 (64.3)	

*: Fisher's exact test (2-sided).

Using the above described cut-off, 8 of 22 dogs (36.4%; 95% CI:17.2%–59.3%) were ELISA positive. The lowest OD value was 0.14, and this dog had a negative PCR but expelled two *E. granulosus* worms after purge (Table 2); in the remaining 7, two were PCR positive (purge was not performed in these two dogs). From the remaining 5 dogs (all PCR negative), only 3 of them had arecoline purge and one dog expelled *E. granulosus* worms. Considering only those dogs with either demonstrated worms after purge (n = 2) or a positive PCR (n = 2), the minimal prevalence of canine echinococcosis in this population is 4/22 (18%; CI:5.2%–40.3%) (Figure 1).

**Table 2:** Evaluations performed in dogs.

Dog ID	ELISA_OD	ELISA_ratio*	Status**	PCR	Purge
30	0.02	0.22	Negative	Not done	Not done
29	0.02	0.22			
28	0.02	0.22			
16	0.03	0.33			
6	0.04	0.44			
24	0.06	0.67			
20	0.06	0.67			
11	0.06	0.67			
10	0.06	0.67			
2	0.06	0.67			
23	0.07	0.78			
19	0.07	0.78			
1	0.07	0.78			
31	0.08	0.89			
5	0.14	1.56	**Positive**	Negative	**Positive**
22	0.21	2.33	**Positive**	**Positive**	Not done
27	0.25	2.78	**Positive**	Negative	Negative
14	0.25	2.78	**Positive**	Negative	**Positive**
17	0.28	3.11	**Positive**	Negative	Negative
21	0.38	4.22	**Positive**	**Positive**	Not done
26	0.62	6.89	**Positive**	Negative	Not done
25	0.72	8.00	**Positive**	Negative	Not done

*: ELISA ratio = OD sample/OD cut-off.

**: Based on a OD cut off<0.09.

**Figure 2:**
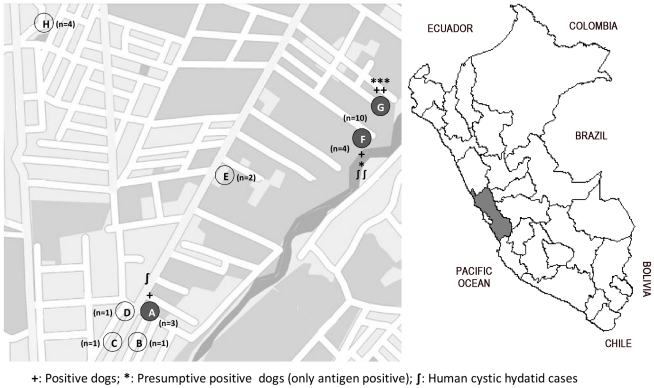
Map of Peru, indicating the geographical localization of Lima and studied abattoirs.

Positive dogs (n = 8) belonged to 3 abattoir centers: Site A, 1/6 (17%); Site F, 2/4(50%); and Site G, 5/10 (50%) (Site Map, Figure 2). Related to the analysis of characteristics of dogs and the abattoir location, (Table 3), in both univariate and multivariate logistic regression analysis the only factor with a positive association with infection was the abattoir location. Dogs from abattoirs close to the river were 36 times more likely to be infected than those from abattoir centers slaughtering animals inside a home (OR = 36; 95%CI: 1.37–934.80; p<0.05).

**Table 3:** Bivariate logistic regression analysis for infection in dogs.

Variable	OR*	95% CI**	p value***
**Age of dogs (months)**			
<12	1	Ref.	Ref.
12–36	3.3	0.27–40.28	0.344
>36	5	0.34–72.76	0.239
**Gender**			
Female	1	Ref.	Ref.
Male	1.66	0.28–9.82	0.572
**Feeding habits of dogs**			
Other	1	Ref.	Ref.
Viscera	0.52	0.05–6.09	0.605
**Localization of abattoir**			
Other	1	Ref.	Ref.
Close to the river	**12.6**	**1.18–133.89**	**0.036**

*: Unadjusted model.

**: CI: Confidence interval.

***: Fisher's exact test (2-sided).

Coproparasitoscopy was performed in 25 fecal samples including 21 that were evaluated by coproELISA: 3 dogs (12%) presented *Taenia sp.* Eggs. From these, one was not evaluated by coproELISA, one was ELISA negative, and one was ELISA positive. Additionally we found *Toxocara sp.* in 16 samples (64%), followed by *Ancylostoma* in 7 (28%), *Isoospora* in 7 (28%) and *Dipilidium sp.* in only 3 (12%) of the samples.

**Figure 3:**
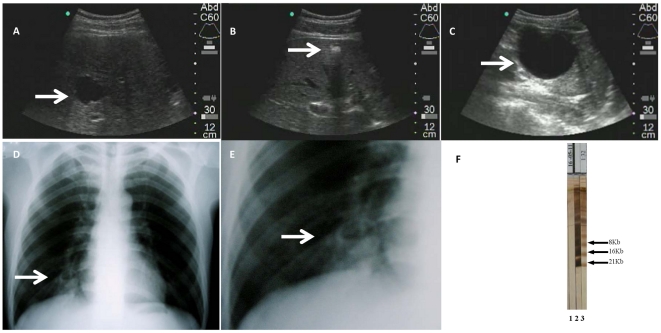
Human cystic hydatid disease. Top row: Abdominal US images of patients with liver hydatid cysts, stages CE1 (A and C) or CE4 (B). Bottom row: Chest X-Rays (D and E) demonstrating a cystic lesion in the cardiophrenic angle, note the presence of air-liquid levels (arrow). (F) EITB result of a patient with liver and lung disease (strip 2), compared to a positive control (strip 3) and a negative control (strip1).

In 6 out of the 8 studied abbattoirs, family members accepted to be evaluated for hydatid infection. From 39 family members in these abattoir centers, 7 were not evaluated (mostly because they were not present at the days of evaluation). Therefore, we analyzed data from 32/39 subjects. Their median age was 24.5 years (range: 3–76), and 16 of them (50%) were male. Ultrasound evaluation found images compatible with CE in 3/32 (9.3%; 95% CI: 2–25; two CE1 cases and 1 CE4 case) (Figure 3). Chest X–rays were performed in 18/32 subjects, and only one (also positive on liver US), had a image compatible with a complicated lung cyst (Figure 3). Finally, serum EITB was performed in 23/32, and only one (the one positive to both liver US and chest X-rays) was seropositive. Therefore the prevalence of human CE among this population was 9.3% (95% CI: 2–25). The three infected individuals were asymptomatic and none presented a history of residence in an endemic area. Two out of these three human cases belonged to abattoir centers where at least one dog was positive (Site A and Site F) (Figure 2).

## Discussion

This study found a high prevalence of canine echinococcosis by coproELISA (8/22, 36%), and also of CHD (9.3%, 3/22), demonstrating autochthonous transmission of *E. granulosus* in Lima, a large metropolis supposedly non-endemic [13]. These findings also confirm the risk of informal, unlicensed abattoirs for urban hydatid disease transmission [6], [12].

Using interviews with the owners of abattoir dogs, we explored putatively associated risk factors reported by other studies such age, sex, and whether dogs were fed viscera [4], [12] [22]. We found no association between these factors and the likelihood of a dog being infected. However, regarding feeding dogs with viscera, we could not directly observe owners' habits so as to verify the information provided during interviews. Additionally, we explored the effect of abattoir location and found that this was the only factor with a positive association with dogs being infected. A tentative explanation is that dogs in abattoirs slaughtering animals close to the river may have more access to infected viscera (people who work in these abattoirs could be using the rivers to discard contaminated viscera). The association between inappropriately discarding viscera and an elevated risk of *E. granulosus* dog infection was previously reported in a study performed among stray dogs that were captured close to abattoirs; authors of that work noted that the high prevalence observed (6%) was associated with the dogs' behavior of scavenging rubbish close to abattoirs [12].

We used primarily the coproELISA results to define infected cases since it is a technique that has some technical and logistic advantages in relation to other techniques e.g. the way to collect sample (in arecoline purge sample collection is laborious and risky); also, coproELISA is faster to perform and requires fewer personnel than the cumbersome, furthermore despite coproELISA performance can be affected due to cross-reaction with antigens of *Taenia sp.* and other helminthes (specificity range 88 to 96%) [16], [17], [22], [23]., the reported sensitivity of coproELISA varies between 76 and 83% [1], [22], [23]. This variation related to the parasite load found in the dog's intestines, with a moderate to high load (>100 parasites) corresponding to a high test result. Additionally; sensitivity of coproPCR seems lower, in a previous study in experimentally infected dogs coproPCR detected 25.9% of *E. granulosus* infected dogs and produced no false positive reactions, while arecoline purgation was 100% specific with a sensitivity of only 64% [18]. Therefore we cannot exclude further cases of dog infection in the copro-ELISA negative animals. On the other hand, from the most conservative standpoint, a minimum of 4 dogs (4/22, 18%; two PCR positive and two purge positive) were infected. We could not calculate the sensitivity of the purge and PCR because of the lack of a gold standard test.

Dogs located near abattoirs are, as any other dogs, usually treated as pets and kept in close contact with families and workers, exposing them to the risk of being infected and developing CHD. Informal, unlicensed abattoirs in urban areas of endemic countries should be a target for control interventions to prevent the appearance of autochthonous cases.
